# Temporal and Spatial Analysis of Negative Emotions in China during the COVID-19 Pandemic

**DOI:** 10.3390/bs14020113

**Published:** 2024-02-03

**Authors:** Yating Ding, Lin Wu, Zijian Peng, Bo Liu

**Affiliations:** 1 School of Sociology, Wuhan University, Wuhan 430072, China; vicky-dyt@whu.edu.cn (Y.D.); wlin@whu.edu.cn (L.W.); pppzj0420@whu.edu.cn (Z.P.); 2 School of Philosophy and Sociology, Jilin University, Changchun 130012, China

**Keywords:** negative emotions, spatial clustering, spatial correlation, Moran’s index, Baidu index

## Abstract

This research aims to explore the spatiotemporal distribution patterns of negative emotions in mainland China during different stages of the COVID-19 pandemic and the external factors influencing this clustering. Using Baidu Index data for 91 negative emotion keywords, a retrospective geographic analysis was conducted across Chinese provinces from 14 October 2019 to 7 July 2022. Four spatial analysis methods (Global Moran’s Index, Local Moran’s Index, Bivariate Global Moran’s Index, and Bivariate Local Moran’s Index) are employed to identify potential clustering patterns and influencing factors of negative emotions at different stages. The results indicate that the COVID-19 pandemic significantly intensified the clustering effect of negative emotions in China, particularly with a more pronounced radiation effect in northwestern provinces. Spatial positive correlations are observed between pandemic-related Baidu indices (pandemic Baidu index, government Baidu index, nucleic acid Baidu index) and negative emotions. These findings contribute to understanding the spatiotemporal distribution characteristics of negative emotions in China post the COVID-19 outbreak and can guide the allocation of psychological resources during emergencies, thereby promoting social stability.

## 1. Introduction

The COVID-19 pandemic is an unprecedented catastrophe that has not only profoundly altered the human way of life but has also exacerbated negative emotions among people [1]. Since the outbreak of the pandemic, depression, boredom, anger, anxiety, and panic have become prevalent negative emotions [2,3,4]. A national survey in China revealed that during the outbreak of the COVID-19 pandemic, 27.9% of the survey participants reported symptoms of depression, and 31.6% exhibited symptoms of anxiety, significantly surpassing the pre-pandemic survey data [5]. Compared to positive emotions, negative emotions exhibit a strong contagion and are prone to forming spatial clusters within a short period [6]. In other words, the level of negative emotions in certain areas becomes significantly higher than in others [7], resulting in more severe collective negative emotional issues [8].

There are significant differences in medical conditions and economic levels across China, so there are also large differences in the spatiotemporal distribution of negative emotions [9,10]. In the early stages of the pandemic, Xu found that individuals located far from Wuhan were more susceptible to related information and felt frightened than residents in the epicenter [11]. Meanwhile, during the mid-term of the pandemic, Deng et al. suggested that strict physical measures, such as enforcing social distancing and home isolation, could reduce offline communication, thereby increasing anxiety and depression [12]. Regions repeatedly affected by the pandemic tend to develop higher resilience against rumors, displaying lower levels of negative emotions [13,14]. However, in non-affected areas, the uncertainty about the future often amplifies concerns about becoming the “next infected person” [15]. Negative emotions may generate a “spillover” effect, further influencing the emotional state of surrounding areas [16]. Individuals may undergo brief emotional reactions in crises, but social emotions are more complex. The accumulation of these momentary emotions over time can give rise to more enduring social and emotional trends [7], especially in prolonged crises where emotional shifts may span multiple stages—from initial shock to gradual adaptation [17]. This complexity requires a longer timeframe for comprehension. Therefore, targeted research on social negative emotions in different temporal and spatial contexts is necessary to better comprehend the dynamic changes in societal emotion. Despite existing research focusing on relatively short time frames, for instance, Ning et al. examined risk perception and negative emotions among Chinese residents from 14 January to 22 February 2020 using online questionnaires [18]. Rossell measured changes in negative emotions among Australian adults from April 1 to 4 April 2020, using social media data [13]. Given the prolonged duration of the COVID-19 pandemic and its enduring impact on human society, we emphasize the need to introduce a longer time frame for a more comprehensive understanding of the evolving societal emotional trends. This exploration not only aids in a deeper understanding of long-term societal adaptation to emergencies but also provides substantial guidance for future psychological health interventions and policy formulations.

Research indicates that the pandemic has brought significant uncertainty to society, encompassing uncertainties in the development trends of the outbreak [19], individual health status [20], and the effectiveness of government policies [21]. According to the Uncertainty Theory, uncertainty serves as a crucial cognitive mechanism that triggers alertness and negative emotions [20,22]. To reduce the sense of uncertainty, individuals tend to seek more information when faced with unknown situations [23]. However, the process of information acquisition and perception may amplify individual risk perception, subsequently increasing negative emotions [24]; therefore, some scholars advocate for heightened attention to infodemic [25]. People’s attention to the pandemic includes two types of information: information about the pandemic itself (including the virus’s transmission trends) and information about how to deal with the pandemic (encompassing government prevention policies and information about nucleic acid testing) [26]. Nevertheless, delays, inconsistencies, and falsehoods in relevant information often aggravate a sense of uncertainty and negative emotions. Firstly, pandemic information updates rapidly, but the accuracy and consistency of that information are not always guaranteed, which often includes unverified data, outdated advice, or direct misinformation [27]. Consequently, this contributes to an increase in anxiety and panic [28]. Secondly, due to the highly dynamic nature of the pandemic, people need timely responses from the government. This implies that policies may undergo frequent changes, even to the extent of being inconsistent. Thus, even when based on the latest authoritative information, it may lead to public feelings of uncertainty and unease [18]. Thirdly, public nucleic acid testing is a crucial tool to determine whether individuals have contracted COVID-19, and the results directly impact personal daily life [29]. Nucleic acid tests are widely used to detect the presence of the virus in individuals. The testing helps identify both symptomatic and asymptomatic cases, allowing for timely isolation and treatment. Once a positive nucleic acid result is detected, individuals and their respective locations may face stricter lockdown measures and stigmatization, leading to more severe negative emotions [30]. Research has indicated the impact of information on the pandemic, government, and nucleic acid tests on negative emotions. However, whether these influences vary across different regions and time periods remains unconfirmed. To fill this gap, we will explore the relationship between external information and negative emotions from a temporal and spatial perspective.

In China, Baidu is the most popular search tool, with over 795 million users, and the user penetration rate of Baidu searches exceeds 90% [31]. Baidu Index is a systematic data-sharing platform based on the Baidu search engine, providing a vast amount of search behavior data. Through the Baidu Index, one can obtain the frequency of user attention to a specific keyword within a specific time period [32]. During the pandemic, due to the unknown nature of the virus and social isolation, people chose to search for relevant information on Baidu and expressed their emotions by asking questions to the search engine, such as “How to deal with anxiety caused by COVID-19?” A higher Baidu index for related keywords indicates a higher level of public attention to a particular event. An increasing number of scholars are using the Baidu Index to study social emotions [33,34], especially negative emotions during the COVID-19 pandemic [31,35,36].

In this study, we use the Baidu Index to obtain data on negative emotions. Specifically, the research conducts a phased analysis of the Baidu index for negative emotions among Chinese internet users using Moran’s Index. Global and Local Moran’s Index are adopted to assess the spatial autocorrelation of negative emotions. Additionally, we utilize Bivariate Moran’s Index to analyze the spatial relationships between negative emotions and influencing factors such as the pandemic Baidu index, government Baidu index, and nucleic acid Baidu index. This approach provides valuable insights for future preparedness in similar health crises, contributing to the maintenance of long-term psychological well-being for individuals and society. Accurately identifying the distribution characteristics of negative emotions is crucial for a deeper understanding of the changing trends in people’s emotions during crisis events and society’s response to disasters. Furthermore, this research can effectively guide the allocation of psychological resources, ensuring timely and targeted support in different regions and periods and meeting future mental health needs.

The specific research questions of our research are as follows:

Q1: During the COVID-19 pandemic, is there spatial clustering of negative emotions at different stages?

Q2: During the COVID-19 pandemic, is there a spatial radiation effect of negative emotions across different provinces?

Q3: During the COVID-19 pandemic, is there a spatial correlation between external factors (such as pandemic information, government information, and nucleic acid testing information) and negative emotions at different stages?

## 2. Materials and Methods

### 2.1. Data Source

This study is a retrospective spatiotemporal analysis of negative emotions in 31 provinces, municipalities, and autonomous regions in mainland China. We obtained provincial Baidu indices of 91 negative emotion keywords and 18 influencing factor keywords from the Baidu Index platform. The reliability of the keywords was assessed by seven individuals, including five doctoral students in social psychology and two experts in social psychology, with a reliability score of 0.856. The data collection period for Baidu Index spans from 14 October 2019 to 7 July 2022.

To ensure the scholarly and everyday applicability of our word selection, this study consulted established emotion vocabularies and widely recognized psychological scales known for their authority and reliability. Furthermore, we maximized the functionality of Baidu Index’s knowledge graph, supplementing it with commonly used daily expressions to ensure our word choices strike a balance between academic precision and alignment with daily language (such 52 hertz means lonely). We first identify 10 categories of emotions by the emotional ontology library of Dalian University of Technology [37], the Chinese version of Self-Rating Anxiety Scale [38], the Chinese version of the Self-rating Depression Scale [39], and the Chinese version of the Kessler Psychological Distress Scale [40], then supplement with specific emotional vocabulary by Baidu Index’s knowledge graph. In the end, 91 keywords were selected (relevant keywords are shown in Appendix A).

Similarly, we have referenced the existing literature and utilized Baidu Knowledge Graph technology to enhance the dictionary of influencing factors [10,35,41]: government, pandemic, and nucleic acid (relevant keywords are provided in Appendix B).

### 2.2. Data Processing

#### 2.2.1. Standardization and Time-Slice Processing

Due to significant differences in population among provinces, we standardized the data by dividing it by the number of Baidu users in each province. We take 20 January 2020 as a crucial time point when the Chinese official media officially announced the occurrence of “human-to-human transmission” of the novel coronavirus and implemented a series of emergency measures. Therefore, this study uses 20 January 2020 as a time division point, labeling data before this date as “pre-pandemic” and after this date as “post-pandemic” to contrast and analyze the emotional impact of the novel coronavirus on Chinese society. 

To identify whether there are phased differences in the sentiment of netizens “post-pandemic”, we perform time-slice processing with a division standard of 180 days. Currently, there is no standardized analysis time window for the spatiotemporal analysis of negative societal emotions. In this study, we choose a 180-day timeframe for the following reasons: Firstly, unlike past sudden events such as heavy rain or typhoons, the COVID-19 pandemic has persisted for several years, indicating a more prolonged impact on society. By opting for a longer timeframe, we aim to gain a more comprehensive understanding of the long-term trends and adaptation processes of societal emotions. Secondly, varying regions exhibit significant differences in the development trends of the pandemic. Additionally, the pandemic situation is not the sole factor influencing negative emotions [42]. Utilizing a 180-day timeframe enables us to capture long-term emotional trends in different regions and better account for other variables that may impact societal emotions, such as external information influences. This approach contributes to establishing a more comprehensive analytical framework to explain the spatiotemporal variations in societal emotions across different regions.

Thus, we categorized the “post-pandemic” data into five stages: the first stage from 20 January 2020 to 16 July 2020; the second stage from 17 July 2020 to 13 January 2021; the third stage from 14 January 2021 to 12 July 2021; the fourth stage from 13 July 2021 to 8 January 2022; and the fifth stage from 9 January 2022 to 7 July 2022.

#### 2.2.2. Moran’s Index Analysis

To identify spatial clustering patterns of negative emotions, we employed a series of spatial statistical methods, including Global Moran’s Index, Local Moran’s Index, Bivariate Global Moran’s Index, and Bivariate Local Moran’s Index. 

Firstly, we used the Global Moran’s Index to assess the overall spatial autocorrelation of negative emotions. The index typically ranges between −1 and 1, with values below 0 indicating spatial dispersion, values above 0 suggesting spatial clustering, and values close to 0 implying an essentially random spatial distribution.

Next, through the Local Moran’s Index, we conducted an analysis of the spatial local clustering patterns of negative emotions in each specific province. This analysis is typically represented in a visual format. The Local Moran index not only allows for local clustering analysis but also includes outlier detection. It is noteworthy that when the Local Moran’s Index of a province is significantly greater than 0, and its intensity of negative emotions is consistent with surrounding provinces, it can be defined as “high-high” adjacent. Conversely, if it is a low value and surrounded by other low-value provinces, it is defined as “low-low” adjacent; a high-value province surrounded by low-value provinces is “high-low” adjacent, while a low-value province surrounded by high-value provinces is “low-high” adjacent. If the local Moran index is less than 0, it usually indicates that the spatial distribution of negative emotions in that area has spatial heterogeneity or an atypical pattern.

Finally, we employ the Bivariate Global Moran’s Index and Bivariate Local Moran’s Index to explore the spatial correlation between negative emotions and their influencing factors. The Bivariate Moran’s Index incorporates independent variables into the Moran’s Index. When the Bivariate Global Moran’s Index is positive, it indicates a spatial positive correlation between the two variables; conversely, it suggests a negative correlation when the index is negative. In the Bivariate Local Moran’s Index, “high-high” adjacent means both variables are above the mean and exhibit positive correlation in a certain area, while “low-low” adjacent signifies the opposite. “high-low” adjacent implies that the independent variable is above the mean, the dependent variable is above the mean, and they exhibit spatial negative correlation, whereas “low-high” adjacent indicates the opposite.

The Global Moran’s Index is calculated as follows:

(1) $$GlobalMoran’s\;I=\frac{\sum_{i=1}^{n}\sum_{j=1}^{n}w_{ij}\left(x_{i}-\bar{x}\right)\left(x_{j}-\bar{x}\right)}{S^{2}\sum_{i=1}^{n}\sum_{j=1}^{n}w_{ij}}$$

In Formula (1) $x_{i}$ is the emotions index value of i province, $\bar{x}$ is the mean value of the emotion index value of 31 provinces, $S^{2}$ is the variance of the emotion index value, $w_{ij}$ is the spatial matrix (we choose the most commonly used adjacent spatial weight matrix).

The Local Moran’s Index is calculated as follows:

(2) $$LocalMoran’s\;I=\frac{\left(x_{i}-\bar{x}\right)}{S^{2}}\sum_{j\neq i}^{n}w_{ij}(x_{j}-\bar{x})$$

The Bivariate Global Moran’s Index is calculated as follows:

(3) $$B-GlobalMoran’s\;I=\frac{\sum_{i=1}^{n}\sum_{j=1}^{n}w_{ij}(x_{i}-\bar{x})(y_{j}-\bar{y})}{S^{2}\sum_{i=1}^{n}\sum_{j=1}^{n}w_{ij}}$$

In Formula (3) $x_{i}$ is the independent variable, $\bar{x}$ is the mean of the independent variable, $y_{j}$ is the dependent variable, $\bar{y}$ is the mean of the dependent variable, and $S^{2}$ is the variance of all samples.

The Bivariate Local Moran’s Index is calculated as follows:

(4) $$B-LocalMoran’s\;I=z_{i}\sum_{j=1}^{n}w_{ij}z_{j}$$

In Formula (4), $z_{i}$ and $z_{j}$ represent the standardized values of the independent and dependent variables, respectively.

## 3. Results

### 3.1. The Spatial Clustering of the Negative Emotion Index at Each Stage

We analyzed the spatial autocorrelation of ten negative emotions across different provinces in China before and after the COVID-19 pandemic and aggregated these emotions to form the Total Negative Emotions (TNE). The results are shown in Table 1.

Before the pandemic, the Global Moran’s index of TNE did not exhibit significant spatial clustering effects. However, under the influence of the pandemic, four out of five stages show significant spatial clustering of TNE, with index values ranging from 0.150 to 0.183. This confirms the spatial clustering of negative emotions in China caused by the COVID-19 pandemic. This clustering is not random but influenced by the proximity of neighboring provinces. For each stage, in the first stage, all ten emotions show significant clustering effects, with “disappointment” (the Global Moran’s index is 0.257) being the most pronounced. In the second stage, the clustering effects of most emotions are further strengthened, except for “sadness”, “disappointment”, and “confusion”, which show weakened effects. In the third stage, only the Global Moran’s index of “disappointment” increases, and other emotions’ indexes decline. In the fourth stage, the clustering effect of TNE is not pronounced, but some emotions still maintain significant levels, such as “sadness”, “loneliness”, “fear”, “disappointment”, and “disgust”. Finally, in the fifth stage, the clustering effect of TNE remains significant, and the Global Moran’s indices of “anxiousness” and “depression” reach their peak.

The Global Moran’s index confirms the clustering effect of negative emotions brought about by the COVID-19 pandemic on Chinese society. It indicates that the closer the geographical locations of different provinces in China, the more closely aligned their levels of negative emotions. The levels of clustering for various negative emotions generally range from 0.088 to 0.257. In the early stage of the pandemic, the clustering effect mainly reflects disappointment, while in the fifth phase, it mainly manifests as anxiousness. Disappointment is more directed towards external factors [43], expecting changes in the external environment and distancing from sources of stress that trigger negative emotions [44]. On the other hand, anxiousness is directed towards oneself, as people are unable to take effective measures to cope with the crisis, leading to a sense of withdrawal [45]. As the pandemic progresses, people gradually realize that the novel coronavirus cannot be eliminated in the short term. Although the virus’s impact shifts from a threat to life to a threat to survival, the cumulative economic damage and psychological torment increasingly result in spatially clustered forms of anxiousness.

### 3.2. Radiation Effect of Negative Emotions

**Figure 1:**
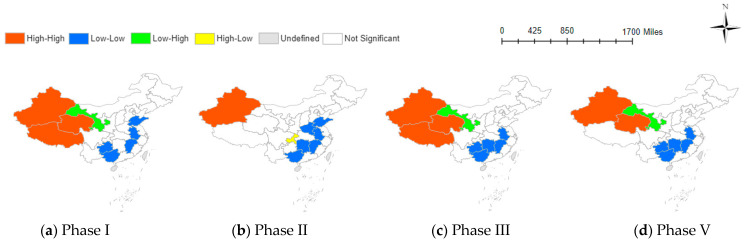
Clustering impact of TNE at various phases.

The Local Moran’s index maps of different stages of TNE drawn by ArcGIS 10.7 are shown in Figure 1. Overall, the emotional clustering in various provinces is mainly characterized by low-low adjacent and high-high adjacent. The southeastern regions of China consistently remain in a state of low-low adjacent, while the northwestern regions fall into the category of high-high adjacent. Xinjiang has the highest occurrence of high-high adjacent among provinces, persisting from the first stage to the fifth stage. Following Xinjiang are Tibet and Qinghai, which exhibit high-high adjacent in the first and third stages, respectively. These three provinces are in the remote northwest of China, with lower levels of economic development and internet penetration. Despite this, negative emotions in these areas surpass the national average, and they exert a positive radiative effect on neighboring provinces.

In contrast, provinces in the east, such as Anhui, and southern provinces, like Jiangxi and Guangxi, consistently show a pattern of low-low clustering in the distribution of negative emotions. This implies that the negative emotions in these three provinces consistently remain at a lower level and exert a negative radiative effect on neighboring provinces. Other provinces exhibiting low-low adjacent, such as Shandong and Guizhou, are also situated in the eastern or southern parts of China.

It is worth noting that Gansu Province is also located in the northwest region, but negative emotions exhibit a low-high clustering phenomenon. Specifically, the negative emotions in Gansu Province are lower than those in the neighboring Xinjiang and Qinghai provinces. Scholars have offered explanations for this phenomenon. Compared to Xinjiang and Qinghai, the capital of Gansu Province is geographically closer to the first epicenter of the pandemic, Wuhan, and is more likely to be influenced by authoritative news. This proximity allows for the acquisition of information, enabling a positive attitude towards future events [45]. Although emotions fluctuate, they generally remain at a positive level. The lowest frequency of occurrence is the high-low clustering type, which is only observed in the second stage in Chongqing. Based on this observation, we referenced the findings of Wang and Tang, indicating a significant correlation between heightened negative emotions in Chongqing compared to other regions and the unemployment rate [46]. As China’s newest directly administered municipality, Chongqing’s economy relies on the tertiary industry. The pandemic has led to substantial fluctuations in the unemployment rate, particularly impacting the tertiary sector. According to the China Statistical Yearbook, Chongqing had a pre-pandemic unemployment rate of 2.62%, notably lower than that of surrounding areas. However, with the pandemic’s onset, the unemployment rate spiked to 4.49%, the highest in the southwestern region. Post-2021, the unemployment rate gradually returned to pre-pandemic levels. The unemployment rate’s fluctuation aligns with the corresponding trend in the accumulation of negative emotions, revealing a highly negative correlation (detailed unemployment rate data in Appendix C).

### 3.3. Spatial Correlation Analysis of Factors Influencing Negative Emotions

Table 2 reveals the dynamic relationship between public negative emotions and three factors: the Government Baidu Index (GBI), the Nucleic Acid Baidu Index (NABI), and the Pandemic Baidu Index (PBI). 

Firstly, the Bivariate Global Moran’s index coefficients of GBI and TNE is not significant before the pandemic. However, during Phases II, III, and IV of the pandemic, these coefficients exhibit spatial significance. 

Similarly, NABI and TNE exhibit significant correlations in Phases I, III, and IV of the pandemic, with correlation coefficients consistently exceeding 0.2.

Furthermore, for PBI and TNE, the Bivariate Global Moran’s index coefficients are significant in all pandemic phases except the first, which also shows a fluctuating upward trend in correlation coefficients.

**Figure 2:**
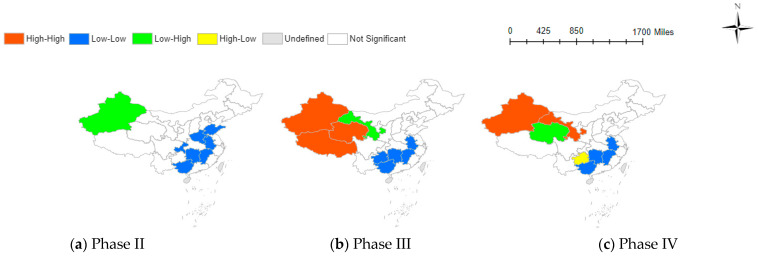
Spatial correlation between GBI and TNE at various phases.

**Figure 3:**
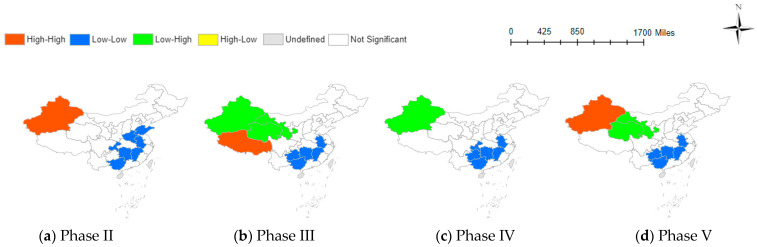
Spatial correlation between NABI and TNE at various phase.

**Figure 4:**
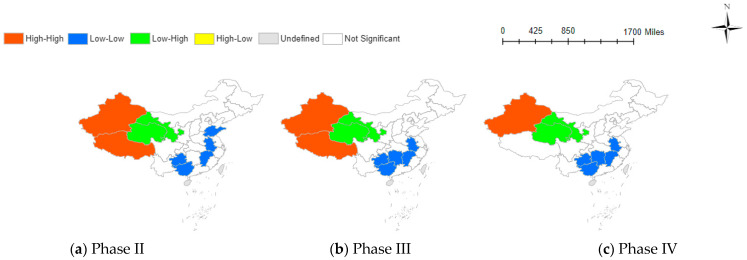
Spatial correlation between PBI and TNE at various phases.

Table 2’s results indicate the varied influence of information factors on social negative emotions across different pandemic stages. Figure 2, Figure 3 and Figure 4 visually illustrate these relationships across provinces, providing a clearer understanding of the intricate dynamics.

#### 3.3.1. The Spatial Correlation Analysis between the GBI and the TNE

The Bivariate Local Moran’s Index maps of GBI and TNE are shown in Figure 2. This correlation is mainly characterized by the low-low adjacent of low GBI and low TNE and the high-high adjacent of high GBI and high TNE. The high-low adjacent of high GBI and low TNE and the low-high adjacent of low GBI and high TNE are concentrated in the western region.

For specific provinces, the eastern and southern provinces show lower levels of government attention in all three phases. However, the northwestern provinces exhibit higher government attention during the third and fifth phases compared to other provinces. GBI is derived from Baidu users actively searching for government-related keywords. From a psychological perspective, external risks reduce people’s confidence in their preferences and choices, making them more likely to trust external actors (government). Consequently, the negative emotions caused by the pandemic increase people’s attention to the government [47]. Therefore, provinces with higher levels of negative emotions, such as Xinjiang, Qinghai, and Tibet, are likely to increase searches for government information.

#### 3.3.2. Spatial Correlation Analysis between NABI and TNE

The Bivariate Local Moran’s Index maps of NABI and TNE are shown in Figure 3. Nucleic acid testing is widely used in China to determine whether individuals are infected with the novel coronavirus. However, the recurrent nature of the COVID-19 pandemic has led to frequent nucleic acid testing, resulting in changing emotional attitudes among Chinese residents towards these tests. 

Examining the specific geographical distribution, southeastern provinces still exhibit a low-low clustering of both low NABI and low TNE. In the northwest region, there are significant spatial variations in correlation patterns. For instance, Xinjiang province transitions from a high NABI and high TNE in the second and fifth stages to a low NABI and high TNE in the third and fourth stages. This phenomenon may be related to the local epidemic situation in Xinjiang. It is observed that when Xinjiang province is at a low-risk level (cumulative confirmed cases ranging from 20 to 199), there is increased attention to nucleic acid information. However, during periods of extremely low risk (cumulative confirmed cases fewer than 19), there is less search activity related to nucleic acid information [11].

#### 3.3.3. Spatial Correlation Analysis of the PBI and TNE

The Bivariate Local Moran’s Index maps of PBI and TNE are shown in Figure 4. The correlation coefficients show minimal variations across different stages. The predominant clusters include the LL type (low PBI-low TNE), LH type (low PBI-high TNE), and HH type (high PBI-high TNE). Like the spatial distribution of other influencing factors, LL is mainly distributed in the eastern and southern provinces of China, such as Shandong, Anhui, Jiangxi, Hunan, Guangxi, and Guizhou. HH is primarily concentrated in Xinjiang and Tibet, while LH is distributed in Gansu and Qinghai. There is significant regional variation in the Baidu index related to COVID-19 in the northwest region, with higher indices in Xinjiang and Tibet and lower indices in Gansu and Qinghai. By the fifth stage, a high COVID-19 search index is only present in Xinjiang, possibly due to its proximity to multiple countries and increased susceptibility to imported cases [48].

## 4. Conclusions and Discussion

Unlike natural disasters, the impact of the COVID-19 pandemic on individuals is more covert. Beyond physical health, the negative effects on people’s mental and emotional well-being deserve greater attention. Exploring the patterns of negative emotional changes in the context of the pandemic can assist health policymakers in adopting more targeted approaches to address potential future issues. This study utilizes the Baidu Index to construct a Chinese Negative Emotion index, analyzing the negative emotional impact of the COVID-19 pandemic on Chinese society to identify spatial clusters of negative emotions at different stages. The study finds a prolonged clustering of negative emotions in Chinese society following the outbreak of the COVID-19 pandemic. The eastern and southern provinces predominantly exhibit low clustering of negative emotions, while the northwestern provinces show high clustering. Before the outbreak, the four phases reflected a clustering effect directed outward in the form of disappointment. In the fifth stage, the clustering effect of anxiousness becomes more apparent. Additionally, the study observes positive spatial correlations between the government Baidu index, nucleic acid Baidu index, pandemic Baidu index, and negative emotions at different stages.

This research emphasizes the evolution of social emotions, using the Baidu Index to comprehensively understand the temporal patterns of negative emotions in major provinces of mainland China. Notably, the southeastern provinces consistently maintained lower levels of negative emotions since the outbreak, while the northwestern provinces, especially Xinjiang, exhibit emotions consistently higher than the national average. In addressing Xinjiang’s unique circumstances, four possible explanations are considered: its unique geographical location, lower GDP, limited economic and medical resources, and the prolonged lockdown leading to a surge in residents’ negative emotions. Among various influencing factors, the study finds that the high levels of negative emotions in the early stages were related to the search volume for pandemic-related topics. However, as the pandemic progressed, the spatial correlation between negative emotions and search volumes for government, nucleic acid, and pandemic information strengthened, especially in remote northwest regions. This suggests that policymakers should provide more information support to alleviate the high levels of negative emotions in northwestern provinces.

## 5. Limitations and Policy Suggestions

The study has limitations, primarily in the choice of data. Provincial-level data are analyzed instead of more detailed city-level or administrative unit data. While provincial data provide an overview of emotional differences, analyzing data at the city or lower administrative levels could offer more nuanced insights for future research. Baidu Index is used to construct the negative emotion index, and though it covers most words, there might be some newly coined terms not included, potentially limiting the analysis. Additionally, there is controversy about the classification of pandemic stages. We adopt a 180-day time slice in this study, which might result in some information being overlooked. Future research could explore alternative time frames. Finally, due to the gender analysis feature of the Baidu Index being available only for keywords with large search volumes, this study does not account for gender-related factors. However, there may be differences in negative emotions experienced by different genders during the pandemic. Future research could explore incorporating gender disparities into consideration.

Based on our research findings, we propose the following three policy suggestions: Firstly, the government should address social negative emotions at different stages, focusing on public disappointment in the early stage of the pandemic and shifting attention to interventions for anxiousness later. Secondly, we suggest strengthening mental health services in remote western areas, accompanied by additional government information support (such as medical information and policy information), to effectively tackle persistent emotional challenges. Lastly, in the face of unforeseen events, ensure transparency and timely communication of information and enhance online management to combat misinformation. These policy recommendations aim to better address societal emotional needs across different stages and regions.

## Figures and Tables

**Table 1:** Spatial agglomeration of different negative emotions.

		Pre-Pandemic	Phase I	Phase II	Phase III	Phase IV	Phase V
TNE	Moran’s I	0.049	0.170 **	0.183 *	0.150 **	0.151	0.148 **
	Z-Value	0.783	3.635	2.047	3.575	1.068	3.365
Sadness	Moran’s I	0.076	0.209 **	0.174 *	0.154	0.164 *	0.111 **
	Z-Value	1.139	3.895	2.033	3.705	1.979	3.182
Anger	Moran’s I	0.045	0.124 **	0.193 *	0.088 *	0.152	0.088 *
	Z-Value	0.750	2.776	2.012	2.512	1.090	2.381
Loneliness	Moran’s I	0.092	0.182 **	0.204 *	0.159 **	0.179 *	0.144 **
	Z-Value	1.147	3.721	2.275	3.582	2.070	3.300
Fear	Moran’s I	0.122	0.101 **	0.123 **	0.116 *	0.193 *	0.148 **
	Z-Value	1.382	2.704	2.610	3.150	2.045	3.641
Anxiousness	Moran’s I	−0.030	0.133 **	0.173 *	0.125 **	0.126	0.232 ***
	Z-Value	0.003	3.068	1.970	3.100	1.407	4.547
Disappointment	Moran’s I	0.108	0.257 **	0.180 *	0.207 **	0.196 *	0.160 **
	Z-Value	1.512	4.237	2.071	4.001	2.211	3.854
Disgust	Moran’s I	0.076	0.112 **	0.222 *	0.110 **	0.210 *	0.212 **
	Z-Value	0.997	2.699	2.217	3.001	2.146	4.400
Helplessness	Moran’s I	0.074	0.145 **	0.199 *	0.148 **	0.182	0.143 **
	Z-Value	0.980	3.347	2.176	3.551	2.049	3.478
Depression	Moran’s I	−0.011	0.133 **	0.159 *	0.105 **	0.145	0.231 **
	Z-Value	0.226	4.136	1.963	3.973	1.890	4.523
Confusion	Moran’s I	0.019	0.168 **	0.138	0.149	0.085	0.088 *
	Z-Value	0.531	3.534	1.691	3.380	1.164	1.972

Significance values: *** *p* < 0.01, ** *p* < 0.05, * *p* < 0.1.

**Table 2:** Spatial clustering of different influencing factors of TNE.

	Influencing Factors of TNE	Moran’s I	Z-Value
Pre-pandemic	GBI	0.034	0.733
Phase I	GBI	0.049	0.672
	NABI	0.031	0.325
	PBI	0.240 **	3.230
Phase II	GBI	0.088 *	1.965
	NABI	0.141 *	2.144
	PBI	0.047	1.303
Phase III	GBI	0.253 **	3.345
	NABI	0.166 *	1.988
	PBI	0.240 **	3.350
Phase IV	GBI	0.075	1.264
	NABI	0.131 *	1.393
	PBI	0.024	0.860
Phase V	GBI	0.119 **	4.114
	NABI	0.202 **	2.538
	PBI	0.221 **	2.770

Significance values: ** *p* < 0.05, * *p* < 0.1.

## Data Availability

Research scholars interested in working with the data used in this study are asked to contact the corresponding author with a short description of the planned study. According to our data-sharing policies, we then require (1) a data management plan, (2) a signed data-sharing and confidentiality agreement, and (3) ethical clearance from an institutional Ethics Committee.

## Appendix A

**Table A1:** Negative emotions words dictionary.

Categories	Emotions	Words
1	Sadness	Sadness, Sorrow, Grief, Unhappiness, Melancholy, Heartache, Pain, Mourning, Gloominess, Dejection, Despondency [37]
2	Anger	Anger, Rage, Fury, Indignation, Resentment, Exasperation, Wrath, Ire [37]
3	Lonely	Loneliness, Solitude, Isolation, Lonesomeness, Autism, Solitary, Misunderstood, Desolate, 52 Hertz, Alone [37]
4	Fear	Fear, Anxiety, Confusion, Nervousness, Terror, Panic, Worry, Unease, Dread [37]
5	Anxiousness	Anxiety, Heartburn, Anxiety, Hair Loss, Anxiety Disorder, Distress, Irritation, Restlessness, Agitation, Vexation, Haggard, Distress, Headache, Very Annoyed [38]
6	Disappointment	Disappointment, Disillusionment, Despair, Frustration, Hopelessness, Defeated [37]
7	Disgust	Disgust, Dislike, Hatred, Loathing, Resentment [37]
8	Helplessness	Helplessness, Wry Smile, Heartbreak, Powerlessness, Speechless, At A Loss, Bewildered, Powerless [37]
9	Depression	Depression, Melancholy, Depression, Insomnia [36,39]
10	Confusion	Doubt, skepticism, Perplexity, Confusion, Bewilderment, Uncertainty, Ignorance, What, Where, How, What Is, How About Curiosity, Wonder, Confusion, Puzzlement, Strange [37]

## Appendix B

**Table A2:** Influencing factors words dictionary.

Categories	Influencing Factors	Words
1	Government	Government, Department, Ministry of Health, Health Bureau, National Health and Family Planning Commission, National Health Commission, Ministry of Health of the People’s Republic of China [41]
2	Pandemic	COVID-19, Virus, COVID-19 Pandemic, Epidemic, COVID, Novel Coronavirus, Pneumonia, Novel Pneumonia, Novel Coronavirus Pneumonia [35]
3	Nucleic Acid	Nucleic Acid, Nucleic Acid Testing [10]

## Appendix C

**Table A3:** Unemployment rate of Chongqing, Sichuan, Hubei, Hunan, Guizhou, and Shaanxi.

Year	Provinces	Unemployment Rate (%)	Sources
2019	Chongqing	2.62	The China Statistical Yearbook of 2020 https://www.stats.gov.cn/sj/ndsj/2020/indexch.htm (accessed on 15 December 2023)
	Sichuan	3.31	
	Hubei	2.24	
	Hunan	2.73	
	Guizhou	3.11	
	Shaanxi	3.23	
2020	Chongqing	4.49	The China Statistical Yearbook of 2021 https://www.stats.gov.cn/sj/ndsj/2021/indexch.htm (accessed on 15 December 2023)
	Sichuan	3.63	
	Hubei	3.35	
	Hunan	2.74	
	Guizhou	3.75	
	Shaanxi	3.63	
2021	Chongqing	2.92	The China Statistical Yearbook of 2022 https://www.stats.gov.cn/sj/ndsj/2022/indexch.htm (accessed on 15 December 2023)
	Sichuan	3.6	
	Hubei	2.99	
	Hunan	2.29	
	Guizhou	4.45	
	Shaanxi	3.47	
